# The effect of *BsmI* (rs1544410) single nucleotide polymorphism of vitamin D receptor (VDR) on insulin resistance in healthy children and adolescents: a cross-sectional study

**DOI:** 10.1186/s12887-023-04503-2

**Published:** 2024-01-17

**Authors:** Ahmad Gholami, Nima Montazeri-Najafabady, Iman Karimzadeh, Mohammad Hossein Dabbaghmanesh, Elnaz Talei

**Affiliations:** 1 Biotechnology Research Center, Shiraz University of Medical Sciences, Shiraz, Iran; 2 Endocrinology and Metabolism Research Center, Nemazee Hospital, Shiraz University of Medical Sciences, Shiraz, Iran; 3 Department of Clinical Pharmacy, School of Pharmacy, Shiraz University of Medical Sciences, Shiraz, Iran

**Keywords:** Insulin resistance, Polymorphism, Vitamin D receptor, Children

## Abstract

The increasing prevalence of metabolic syndrome, type 2 diabetes, and insulin resistance are driven by complex interactions between genetic and environmental factors. One of the single nucleotide polymorphisms (SNPs) in the VDR gene associated with vitamin D levels is the rs1544410 SNP. This study examined the association of the rs1544410 polymorphism with insulin resistance to predict and screen for possible association with type 2 diabetes and target these individuals for appropriate treatment. This cross-sectional study examined 270 children and adolescents aged 9 to 18 years. Anthropometric and biochemical parameters were determined. Insulin resistance/sensitivity was determined using Quicki, HOMA-IR, MacAuley, Revised MacAuley, Bennetts, FIRI and insulin-to-glucose ratio. The BsmI single nucleotide polymorphism (rs1544410) was determined using the PCR-RFLP method after extracting DNA from peripheral blood collected from fasted subjects, and the resulting data were analyzed using SPSS software and statistical tests.

According to linear regression analysis, a significant difference was found in Insulin to glucose ratio, FIRI and HOMA-IR indices between Bb / bb and BB genotypes and it was observed that individuals with BB genotype polymorphism of *BsmI* vitamin D receptor gene, after Adjustment of age, sex, BMI are at greater risk for insulin resistance and type 2 diabetes.

This study demonstrated that those with the BB genotype of VDR *BsmI* polymorphism were at higher risk for insulin resistance and developing type 2 DM.

## Introduction

The metabolic syndrome refers to the classification of common risk factors for cardiovascular disease. This syndrome is also known as “insulin resistance syndrome”. An important part of its pathophysiology depends on the resistance to the metabolic effects of insulin [1]. These risks progressively initiate in childhood and adolescence and may be accompanied with chronic disease in adulthood.

A major cause of childhood insulin resistance is a common pattern of lipid distribution characterized by increased lipid deposition in insulin-responsive tissues such as the liver, skeletal and visceral tissues. This lipid deposition pattern is concomitant with the infiltration of immune system cells into intraperitoneal tissues, causing systemic and low-grade inflammation in insulin-resistant obese children and adolescents [1, 2].

Insulin resistance leads to increased fasting blood glucose and pre-diabetes (impaired glucose tolerance), with a progressive decline in both insulin secretion and insulin sensitivity [3]. Both genetic and environmental factors contribute to the pathogenesis of insulin resistance [4]. Impaired insulin signaling and several post-receptor intracellular defects, including impaired glucose transport, glucose phosphorylation, and decreased glucose oxidation and glycogen synthesis, underlie the decreased insulin-stimulated glucose uptake in skeletal muscle. Although the precise mechanisms of insulin resistance in skeletal muscle are not entirely understood, increased intramyocyte fat content and fatty acid metabolites play an important role in the development of insulin resistance in skeletal muscle [5]. An additional factor that connects obesity to increased insulin resistance is systemic inflammation. The diffusion (infiltrate) of the immune system cells (mainly macrophages) into the subcutaneous and intra-abdominal lipid depots have the potential to induce local and systemic inflammatory activation [2].

Animal studies have shown that vitamin D is a basic factor, necessary for normal insulin secretion. Vitamin D reduces insulin resistance probably through its effect on calcium and phosphorus metabolism and through up regulation of the insulin receptor gene [6].

Researchers also discovered that the VDR (vitamin D receptor) regulates vitamin D levels and calcium metabolism in the body, which are associated with endocrine dysfunction and insulin resistance [6]. The vitamin D receptor is a member of the nuclear receptor superfamily of transcriptional regulators, which can be found in most tissues and body cells [7, 8]. 1, 25 (OH)_2_ D is the main ligand for the vitamin D receptor (VDR). Ligand-activated VDR presumably modulates the expression of many genes [8]. The VDR gene, which is located on chromosome 12q13.1, composed of 14 exons and has an extensive promoter region adept of making multiple tissue-specific transcripts [9]. The 3’ untranslated region of the human VDR gene because of its functional role in regulating gene expression, particularly through regulation of messenger RNA (mRNA) stability and protein translation efficiency has been receiving the most attention recently. This region plays a role in regulating gene expression, particularly through regulation of messenger RNA (mRNA) stability and protein translation efficiency. *FokI*, *BsmI*, *ApaI* and *TaqI* are the four common polymorphisms of the VDR gene which have been identified and designated in detail previously [9].

rs1544410; One of the SNPs in the vitamin D receptor gene related to vitamin D levels is located in the intron region (intron 8 near the 3’ end). This SNP (Single Nucleotide Polymorphism) is a restriction fragment length polymorphism for the restriction endonuclease *BsmI*, also known as the *BsmI* polymorphism [10, 11].

Previous studies showed that participants with the bb genotype for the *BsmI* polymorphism had higher HOMA-IR (Homeostatic Model Assessment for Insulin Resistance) levels compared with participants with BB and Bb genotypes only after further adjustment for calcium and vitamin D use [12]. Rivera-Leon et al. did not observe any association between *TaqI* and *ApaI* VDR gene polymorphisms in T2DM participants [13]. Wang et al. found that VDR *BsmI* polymorphism may enhance the susceptibility to T1DM in East Asians [14]. Mackawy and Badawi described higher TC (Total cholesterol), TG (Triglyceride), LDL-C (Low density lipoprotein) and lower HDL (High density lipoprotein) levels in T allele carriers as well lower vitamin D levels in homozygous recessive VDR 2,228,570 C > T (*FokI*) and VDR 1,544,410 A > G (*BsmI*). Vit D levels were lower in homozygous recessive VDR 2,228,570 C > T (*FokI*) and VDR 1,544,410 A > G (*BsmI*) [15].

Alterations in vitamin D receptors may contribute to type 2 diabetes through at least four different pathophysiological pathways:(i) extracellular calcium metabolism, (ii) intracellular calcium in adipocytes, and (iii) insulin secretion regulation, and (iv) cytokine metabolism [16]. Vitamin D has also been suggested to prevent the expression of IL-2 produced by activated human T lymphocytes [17]. Thus, inhibition of vitamin D binding to its receptors and subsequent signaling may alter the profile of cytokine secretion. Patients with type 2 diabetes exhibit subtle changes in glucose metabolism long before disease onset, allowing the identification of etiological or contributing genetic factors early in the disease process [9].

Because the prevalence of type 2 diabetes is increasing worldwide and current methods of treating diabetes are inadequate, the most effective way to reduce the burden associated with type 2 diabetes is to prevent it [18]. As we know, both genetic and environmental factors contribute in pathogenesis of insulin resistance [4]. Subtle alterations in glucose metabolism are apparent long before the onset of type 2 diabetes, and identification of the genetic factors that contribute to its etiology or development [9], make it possible to provide better and personalized preventive measure based on genetic information. So that this study aimed to examine the association of the vitamin D receptor *BsmI* rs1544410 polymorphism with insulin resistance in Iranian children and adolescent to predict and screen for possible association with type 2 diabetes and target these individuals for appropriate treatment.

## Subjects and methods

### Study population

270 children and adolescents (9 to18) years old, recruited in this study (136 girls and 134 boys) who were randomly selected and enrolled for genotype analysis. They were picked from Kawar (an urban area located 50 km east of Shiraz, the capital city of the Fars province in the south of Iran. The participants consisted of randomly selected children aged at least 9 years or older, as well as adolescents attending elementary, guidance, or secondary school. Random, systematic sampling was applied to gather the sample group of that survey. An age-stratified systematic random sample of 7.5% was used, and 500 children (250 girls and 250 boys) were selected. We use simple, random sampling to generate random numbers with SPSS software and we selected 270 children from that survey population to do the genetic study. Children and adolescents with systemic impairment (e.g. thyroid complications, diabetes, renal problem, adrenal deficiency), history of developed postponed puberty, using any medications (e.g. anticonvulsants or steroids), were omitted from the study as reported in our recent publication [19]. Ethical approval for this study was obtained from the ethics committee of Shiraz University of Medical Sciences. A written informed consent was obtained from participants and their parents.

### Anthropometric and pubertal assessments

The body weight of the participants was measured using a standard scale (Seca, Germany) to the nearest 0.1 kg, and height of the children was measured using a wall-mounted meter to the nearest 0.5 cm [19].

BMI was calculated by this formula: BMI (kg/m^2^) = weight (kg) / [height (m)]^2^ [19].

An endocrinologist assessed the children’s pubertal stage according to the Tanner pubertal stages. Pubertal stage was evaluated according to the Tanner standard classification during the visit for the Dual-energy X-ray absorptiometry (DEXA) scan. Children with Tanner stages of 1 were considered as prepubertal, 2 and 3 as early pubertal, and children with Tanner stages of 4 and 5 were defined as pubertal.

### Biochemical parameters

Fasting venous blood (after at least 8 h of fasting) was drawn from the participants by trained physicians in the Shiraz Endocrinology Research Center. Serum total cholesterol, high-density lipoprotein (HDL-C), fasting blood sugar (FBS), and triglyceride (TG) concentrations were quantified by enzymatic reagents (Biosystems, Barcelona, Spain) with an A-25 Biosystems auto-analyzer. The serum insulin concentration was measured with a radioimmunoassay kit (IZOTOP, Budapest, Hungry). Low-density lipoprotein (LDL) concentrations were calculated from the quantified levels of TG, HDL-c, and TC according to the Friedewald equation as described previously [20]. The coefficient of variation (CV%) for TG, TC, and HDL methods was 1.7–2.6%, 1–1.9.5%, and 1.3–1.5%, respectively.

LDL-c (mg/dL) = TC (mg/dL) − HDL-c (mg/dL) − TG (mg/dL)/5 [20].

Insulin resistance/sensitivity assessment:

Insulin resistance/sensitivity was assessed by the QUICKI, HOMA-IR, insulin-to-glucose ratio, McAuley, revised McAuley, FIRI, and Bennett’s indices and calculated using the bellow formulas [21]:

QUICKI: 1 / log (glucose mg/dL) + log (insulin µU/mL) [21].

HOMA-IR: (fasting insulin [microunits per milliliter] × fasting glucose [millimoles per liter]) / 22.5 [21].

Insulin-to-glucose ratio: Insulin (µU/mL) / glucose (mmol/L) [21].

McAuley: exp [2.63–0.28ln (insulin) − 0.31ln (triglyceride)] [21].

Revised McAuley: exp [3.29–0.25ln (insulin) – 0.22 ln (BMI) – 0.28ln (triglyceride)] [21].

FIRI: Insulin (µU/mL) × glucose (mmol/L) /25 [21].

Bennett’s index: 1 / log [glucose (mmol/L)] × log [insulin (µU/mL) [21].

### Genotyping

Genotyping was described in our previous study [22]. In brief, genomic DNA was extracted from the blood samples, using the QIAamp blood kit (Qiagen, Hilden, Germany), according to the manufacturer’s instructions. *BsmI* (rs1544410) single nucleotide polymorphism (SNP) was identified using PCR/RFLP (polymerase chain reaction/restriction fragment length). PCR amplification was performed using the following primers:

Forward 5’TGAAGGGAGACGTAGCAA 3’;

Reverse 5’ACCTCATCACCGACATCA 3’.

The details of PCR conditions were mentioned in the following table (Table 1).

**Table 1:** PCR program to amplify the rs1544410 fragment (BsmI) in the VDR gene

Number of cycles	steps	Temperatures (centigrade)	Time (second)
1	Pre-denaturation	°94	300
35	Denaturation	°94	35
	Annealing	°52	30
	Extension	°72	30
1	Final extension	°72	300

Steps 2, 3 and 4 were performed for about 35 cycles

After PCR, RFLP was performed by *BsmI* restriction enzymes. Digested PCR outcomes were determined on 2% agarose gel and detected by a UV transilluminator.

### Statistical analysis

Descriptive data were expressed as mean and standard deviation. All variables were analyzed for Normal distribution by Kolmogorov-Smirnov test. To evaluate the differences between the genotype groups using t-test and ANOVA for data with a normal distribution, and Mann–Whitney and Kruskal–Wallis tests for data with non-normal distribution. Allelic frequencies were estimated by gene counting and genotypic distribution of polymorphisms, and tested for Hardy-Weinberg equilibrium (HWE), by Chi-square analysis.

Linear regression analysis was used to observe the association between VDR polymorphisms and insulin resistance indices, in the dominant and recessive genetic models adjusted for age, sex, BMI, in 3 statistical models. Model 1 was adjusted for age, Model 2 for age and sex, Model 3 for age, sex and BMI. The relationship between different genotypes of rs1544410 (*BsmI*) polymorphism in vitamin D receptor gene was determined using odds ratio (OR) with 95% confidence level (CL) calculated by linear regression. The minor allele of SNP was considered as the reference allele. Analyses were conducted by Package for the Social Science SPSS software for Windows, version 22. *P* values less than 0.05 were considered statistically significant.

## Results

### Characteristics of the study population

This study included 270 healthy Iranian children (136 girls, 134 boys) aged 9–18 years. The means and SDs/medians and interquartile range of clinical features of the participants are shown in Table 2.

**Table 2:** General characteristics, biochemical parameters and insulin resistance indices of the study population

Variables	Mean ± SD	Median (interquartile range)
**General characteristic**		
Age	------	14 (5)
Height (cm)	15.04 ± 154.48	------
Weight (Kg)	------	43 (21)
BMI (Kg/m^2^)	------	17.2 (4.1)
**Biochemical parameters**		
Cholesterol (mg/dL)	------	150 (39)
HDL (mg/dL)	------	45.30 (17.3)
TG (mg/dL)	------	53 (51)
LDL (mg/dL)	------	93.3 (32)
Fasting blood sugar (mg/dL)	12.5 ± 78.87	------
Insulin (mg/dL)	------	8.1 (4)
**Insulin resistance indexes**		
QUICKI-index	------	0.36 (0.03)
HOMA-IR	------	1.53 (0.89)
Insulin-to-glucose ratio	------	1.8 (1.09)
McAuley	------	2.3 (0.92)
Revised McAuley	------	2.84 (1.04)
FIRI	------	1.38 (0.8)
Bennett’s index	------	1.76 (0.75)

### Genotype frequencies and distribution of VDR polymorphisms *BsmI* (rs1544410)

After genotyping, we identified 49 (18.15%) BB, 128 (47.45%) Bb, and 93 (34.4%) bb participants. The allele frequency of the VDR *BsmI* polymorphism was 41.85% B (n = 226) and 58.15% b (n = 314). Genotype counts and frequencies of the VDR polymorphism are presented in Table 3. The distribution of genotypes was in compliance with the Hardy-Weinberg equilibrium (*P* = 0.67). There were no significant differences between allele distribution and sexes (*P* = 0.503).

**Table 3:** Genotype counts and frequencies of the rs1544410 polymorphism

	Boys	Girls	Total	*p* value
**Genotypes**				
BB	24 (17.9%)	25 (18.4%)	49 (18.15%)	
Bb	68 (50.8%)	60 (44.1%)	128 (47.45%)	0.502
bb	42 (31.3%)	51 (37.5%)	93 (34.4%)	
**alleles**				
B	116 (43.3%)	110 (40.4%)	226 (41.85%)	0.503
b	152 (56.7%)	162 (59.6%)	314 (58.15%)	

### *BsmI* (rs1544410) polymorphism effects on insulin indices

The analysis of *BsmI* genotype showed no significant effect on fasting blood glucose, serum insulin concentration, QUICKI, Bennett’s index, HOMA-IR, FIRI, insulin-to-glucose ratio, and McAuley and revised McAuley indices (Table 4). However, the Bb carriers had lower levels of FBS, serum insulin concentration, HOMA-IR, FIRI, and insulin-to-glucose ratio.

**Table 4:** Effect of the *BsmI* (rs1544410) polymorphism on insulin function indices

Data	Genotype			*p* values
	BB	Bb	bb	
	Mean (SD)			
Fasting blood sugar	78.77 (12.05)	78.04 (12.9)	78.33 (12.38)	0.94
Insulin	10.47 (7.06)	8.42 (5.17)	8.95 (4.78)	0.106
QUICKI	0.35 (0.03)	0.36 (0.04)	0.36 (0.04)	0.336
HOMA-IR	2.07 (1.84)	1.71 (1.22)	1.75 (1.11)	0.333
Insulin-to-glucose ratio	2.34 (1.35)	1.93 (1.12)	2.01 (1)	0.126
McAuley	2.30 (0.66)	2.31 (0.69)	2.46 (0.93)	0.352
Revised McAuley	2.86 (0.76)	2.86 (0.79)	3.01 (1.06)	0.462
FIRI	1.92 (1.57)	1.50 (1.06)	1.63 (1.03)	0.125
Bennett’s index	1.84 (0.73)	1.88 (0.84)	2.09 (2.67)	0.635

### Association of rs1544410 polymorphism with insulin resistance indices

The effect of VDR *BsmI* polymorphisms on insulin resistance indices of the categorized groups in two genetic models (Dominant and Recessive) is presented in Table 5. Linear regression analysis results demonstrated significant association between HOMA-IR in the recessive genetic model 3 [*P* = 0.046, OR (CI 95%): -0.129 (-0.448, -0.004)] and FIRI in the recessive genetic models [model 1: *P* = 0.048, OR (CI 95%): -0.125 (-0.378, -0.002); model 2: *P* = 0.031, OR (CI 95%): -0.132 (-0.383, -0.018); model 3: *P* = 0.015, OR (CI 95%): -0.148 (-0.409, -0.045)] and insulin-to-glucose ratio in the recessive genetic models [model 1: *P* = 0.031, OR (CI 95%): -0.134 (-0.377, -0.019); model 2: *P* = 0.026, OR (CI 95%): -0.137 (-0.381, -0.024); model 3: *P* = 0.011, OR (CI 95%): -0.153 (-0.404, -0.053)]. No significant association was found between other insulin resistance indices (QUICKI, McAuley, revised McAuley and Bennett’s index) and genetic models of VDR *BsmI* polymorphisms.

**Table 5:** Linear regression analysis for association of insulin resistance indices in 2 genotype groups in 3 models. *P* values less than 0.05 are shown in bold

**HOMA-IR**						
Data	Model 1		Model 2		Model 3	
	OR (CI 95%)	*p* value	OR (CI 95%)	*p* value	OR (CI 95%)	*p* value
*BsmI* (rs1544410)						
Dominant (BB + Bb) Vs bb	-0.033 (-0.228, 0.136)	0.620	-0.026 (-0.212, 0.141)	0.694	-0.036 (-0.226, 0.127)	0.581
Recessive (Bb + bb) Vs BB	-0.111 (-0.420, 0.035)	0.097	-0.116 (-0.421, 0.020)	0.074	-0.129 (-0.448, -0.004)	**0.046**
**FIRI**						
	Model 1		Model 2		Model 3	
	OR (CI 95%)	*p* value	OR (CI 95%)	*p* value	OR (CI 95%)	*p* value
Dominant (BB + Bb) Vs bb	0.001 (-0.151, 0.153)	0.992	0.009 (-0.136, 0.158)	0.884	-0.006 (-0.154, 0.139)	0.921
Recessive (Bb + bb) Vs BB	-0.125 (-0.378, -0.002)	**0.048**	-0.132 (-0.383, -0.018)	**0.031**	-0.148 (-0.409, -0.045)	**0.015**
**Insulin-to-glucose ratio**						
	Model 1		Model 2		Model 3	
	OR (CI 95%)	*p* value	OR (CI 95%)	*p* value	OR (CI 95%)	*p* value
Dominant (BB + Bb) Vs bb	-0.023 (-0.343, 0.235)	0.713	-0.019 (-0.333, 0.243)	0.756	-0.041 (-0.380, 0.185)	0.497
Recessive (Bb + bb) Vs BB	-0.134 (-0.377, -0.019)	**0.031**	-0.137 (-0.381, -0.024)	**0.026**	-0.153 (-0.404, -0.053)	**0.011**
**QUICKI**						
	Model 1		Model 2		Model 3	
	OR (CI 95%)	*p* value	OR (CI 95%)	*p* value	OR (CI 95%)	*p* value
Dominant (BB + Bb) Vs bb	0.021 (-0.005, 0.007)	0.746	0.015 (-0.005, 0.006)	0.813	0.028 (-0.004, 0.007)	0.661
Recessive (Bb + bb) Vs BB	0.096 (-0.002, 0.012)	0.135	0.100 (-0.001, 0.013)	0.111	0.113 (-0.001, 0.013)	0.071
**Bennett’s index**						
	Model 1		Model 2		Model 3	
	OR (CI 95%)	*p* value	OR (CI 95%)	*p* value	OR (CI 95%)	*p* value
Dominant (BB + Bb) Vs bb	0.066 (-0.103, 0.341)	0.293	0.068 (-0.101, 0.344)	0.283	0.073 (-0.093, 0.356)	0.251
Recessive (Bb + bb) Vs BB	0.036 (-0.199, 0.359)	0.573	0.034 (-0.202, 0.356)	0.585	0.037 (-0.199 ,0.366)	0.561
**McAuley**						
	Model 1		Model 2		Model 3	
	OR (CI 95%)	*p* value	OR (CI 95%)	*p* value	OR (CI 95%)	*p* value
Dominant (BB + Bb) Vs bb	0.095 (-0.025, 0.181)	0.136	0.093 (-0.026, 0.179)	0.145	0.114 (-0.008, 0.194)	0.070
Recessive (Bb + bb) Vs BB	0.041 (-0.087, 0.171)	0.525	0.043 (-0.086, 0.173)	0.506	0.051 (-0.075, 0.181)	0.414
**Revised McAuley**						
	Model 1		Model 2		Model 3	
	OR (CI 95%)	*p* value	OR (CI 95%)	*p* value	OR (CI 95%)	*p* value
Dominant (BB + Bb) Vs bb	0.085 (-0.037, 0.196)	0.179	0.083 (-0.039, 0.194)	0.189	0.111 (-0.007, 0.214)	0.067
Recessive (Bb + bb) Vs BB	0.030 (-0.112, 0.183)	0.634	0.033 (-0.109, 0.186)	0.608	0.051 (-0.081, 0.200)	0.403

## Discussion

This cross-sectional study examines *BsmI* polymorphisms in the vitamin D receptor gene and metabolic parameters and insulin resistance indices (HOMA-IR, QUICKI, insulin-to-glucose ratio, McAuley, McAuley revised, FIRI, and Bennett’s index) in Iranian healthy children and adolescents aged 9 to 18 years. In the current study, we observed insignificant variance in genotype distribution and allele frequency between the boys and girls. Also, the results demonstrated consistent associations between the VDR-BsmI polymorphism and HOMA-IR, insulin-to-glucose ratio and FIRI insulin resistance index.

After genotyping we identified 18.15% BB, 47.45% Bb, and 34.4% bb participants. The frequency of alleles in the VDR *BsmI* was 41.85% allele B and 58.15% allele b. Oh and Barrett-Connor showed that prevalence of glucose intolerance (IFG and IGT) was significantly higher in nondiabetic persons with aa genotype compared with those with Aa and AA genotypes of *ApaI* polymorphism.

In the case-control study by Hoseinkhani et al. 99 gastric cancer cases and 100 healthy subjects as controls participated, the b allele frequency was 27.3% in the case group and 29.5% in the control group [23]. In a study of 230 Polish females (110 with recurrent miscarriages and 120 repeatedly enrolled age-matched healthy females with at least two full-term pregnancies and with no history of miscarriages) who were in reproductive age, the allele frequency of *BsmI* polymorphism in the case group was 54.1% for Allele G (b) and 45.9% for allele A (B) and in the control group, 63.7% for allele G (b) and 36.2% for allele A (B). Similar to the findings of our study they found that b allele was dominant in their population. They also observed significant variances in genotype distributions and allele frequencies between case and control groups (GG vs. GA and AA, *p* = 0.036; G/A, *p* = 0.035, respectively) [24].

In this study, we found consistent associations between the VDR-BsmI polymorphism and HOMA-IR, insulin-to-glucose ratio and FIRI insulin resistance index. Regression analysis showed significant differences in HOMA-IR, insulin-to-glucose ratio and FIRI between BB and Bb/bb genotypes (recessive model). Adolescents with the BB genotype of VDR *BsmI* were observed to be associated with an increased risk of insulin resistance compared with the bb/Bb genotype. The HOMA model has been shown to be a vigorous clinical and epidemiological instrument for measuring insulin resistance [25]. Low levels of HOMA-IR designate higher insulin sensitivity, so high levels of HOMA-IR specify low insulin sensitivity, or indeed insulin resistance [26]. Based on Reaven study, reducing the sensitivity of tissue to insulin leads to decreased uptake of glucose in the periphery and increased production of glucose in the liver.

The pancreas enhances secretion of insulin to maintaining normal plasma glucose level, resulting in chronically elevated plasma insulin and normal or elevated (raised) plasma glucose [27].

Therefore, the ratio may destroy the information obtained from fasting insulin and glucose in whom insulin and glucose rise simultaneously, albeit the latter often still within the “normal” range. This led to the introduction of a fasting insulin resistance index (FIRI) consisting of insulin and plasma glucose (normalized to an expected glucose of 5 mmol/L and insulin of 5 mU/L to give a reference range centered around unity). Although HOMA-IR is closely related to insulin resistance, it has been used mostly in small studies. The expression of the FIRI index as the product of fasting glucose and insulin provides a tangible concept of insulin resistance syndrome as it applies to patients. This parameter helps to identify pre-diabetic states in large clinical or epidemiological studies [28]. It seems that recruiting healthy children and adolescents in this study may be the main reason for significant association of HOMA-IR and non-significant association of other insulin resistance indices with VDR polymorphism. HOMA-IR is mostly used in small populations for identifying pre-diabetic stages, while other insulin resistance indices were used in patients with confirmed diabetes.

Vitamin D receptors are expressed in pancreatic beta cells, and VDR polymorphism presence may affect insulin secretion [7].

Some single nucleotide polymorphisms (SNPs) that may be associated with vitamin D levels are localized to the vitamin D receptor gene. The VDR gene plays a central role in regulating the vitamin D pathway and regulating hormone-responsive genes. Interestingly, vitamin D receptor gene is expressed in adipocytes and pancreatic beta cells and are associated with the direct effects of vitamin D on adipocyte differentiation and metabolism, or the indirect effects through regulation of insulin secretion [10]. rs1544410 (*BsmI*) is a restriction fragment length polymorphism of the restriction endonucleases *BsmI* [29]. It has been hypothesized that strong linkage disequilibrium with polyadenosine (poly (A)) microsatellite repeats in the 3’ untranslated region may affect the translational activity of VDR [10, 22].

The association of vitamin D receptor gene polymorphisms with insulin resistance and type 2 diabetes has been investigated in many studies, but the results are inconsistent across different populations. Han et al. conducted a meta-analysis of the association between vitamin D receptor gene variant and insulin resistance related diseases in 28 articles in which 9,232 individuals participated that 5193 of them had insulin resistance related disease. The evidence from that meta-analysis indicated an association between VDR-*BsmI* variants and MetS. In contrast to our results, they support that VDR-BsmI variant G(b) allele may be a susceptibility marker for MetS [6].

Speer et al. stated that postprandial serum C-peptide levels of BB genotypes were significantly greater in the diabetic and obese patients. They concluded that polymorphisms in the VDR receptor gene may play a role in the pathogenesis of type 2 diabetes by affecting the secretory capacity of beta cells [30]. Similar to our results, Ortlepp et al. also found that BB carriers of VDR *BsmI* polymorphism were at greater risk for developing type 2 DM, and also proposed that vitamin D is involved in the pathogenesis of type 2 DM [16]. Our results indicated that based on HOMA-IR, individuals with BB carrier had lower insulin sensitivity compared to Bb/bb carriers. In another study, Ortlepp et al. concluded that Vitamin D receptor B alleles are associated with blunted calcium absorption, more rapid bone loss, decreased bone density, elevated parathyroid hormone and type 2 diabetes in gene carriers, and fasting blood glucose levels in healthy young men. This study showed that the genetic factors of type 2 DM affect the pathogenesis decades before the onset of the disease, regardless of physical activity [31]. Rahmadhani et al. showed a significant difference between the carriers of the A allele of *BsmI* and non-carriers and the risk of insulin resistance in patients with concurrent vitamin D deficiency [10].

This study has some advantages; this is the former study to examine the effect of *BsmI* and insulin resistance in healthy Iranian children and adolescents with different insulin resistance indices.

Based on the limitations we can point to the relatively small sample size, which could affect statistical power to detect association of *BsmI* variants with insulin resistance. In addition, participants registered in the present study were nominated from a cohort study from the south of Iran and may not characterize the general population of Iranian children. The larger sample size would help to lower the variance in the findings and enhance the power of the experiment. To understand the correlation between *BsmI* polymorphism and insulin resistance, and also in order to verify our results, more studies are needed in Iran and other demographic and racial groups. Another limitation of this study is the impossibility of using the direct sequencing technique to confirm the results of different genotypes of *BsmI* polymorphism, due to financial problems and time constraints.

## Conclusion

This study presented the effect of VDR *BsmI* polymorphism on insulin resistance in children and adolescents and showed that adolescents carrying the BB genotype of VDR *BsmI* were associated with increased risk of insulin resistance compared to the bb/Bb genotypes.

## Data Availability

The datasets generated during and analyzed during the current study are not publicly available due to [according to protect the patient information] but are available from the corresponding author on reasonable request.
